# When does activating diversity alleviate, when does it increase intergroup bias? An ingroup projection perspective

**DOI:** 10.1371/journal.pone.0178738

**Published:** 2017-06-05

**Authors:** Melanie C. Steffens, Gerhard Reese, Franziska Ehrke, Kai J. Jonas

**Affiliations:** 1 Department of Social, Environmental, and Economic Psychology, Faculty of Psychology, University of Koblenz-Landau, Landau, Germany; 2 Department of Work and Social Psychology, Faculty of Psychology and Neurosciences, Maastricht University, Maastricht, The Netherlands; Goethe-Universitat Frankfurt am Main, GERMANY

## Abstract

The question how intergroup bias can be alleviated is of much theoretical and practical interest. Whereas diversity training and the multiculturalism ideology are two approaches prominent in practice, most theoretical models on reducing intergroup bias are based on social-identity theory and self-categorization theory. This social-identity perspective assumes that similar processes lead to intergroup bias in very different intergroup contexts if people identify with the respective social groups. A recent prominent model based on these theories is the ingroup-projection model. As this model assumes, an ingroup’s norms and standards are applied to outgroups included in a common superordinate category (this is called ingroup projection). Intergroup bias results because the outgroup fulfils these norms and standards less than the ingroup. Importantly, if the diversity of the superordinate category is induced as the norm, ingroup projection and thus intergroup bias should be reduced. The present research delineates and tests how general this process is. We propose that ingroup prototypicality is not only an outcome variable, as the ingroup-projection model originally assumes, but can also be an important moderator. We hypothesize that for members considering their ingroup highly prototypical (“pars pro toto”, large majorities), the superordinate group’s diversity may question their ingroup’s position and thus elicit threat and intergroup bias. In contrast, for members who consider their group as less prototypical (one among several, or “una inter pares” groups), activating diversity should, as originally assumed in the ingroup-projection model, reduce intergroup bias. Three experiments (total *N* = 345) supported these predictions in the contexts of groups defined by gender or nationality. Taken together, the ingroup-projection model can explain under which conditions activating superordinate-category diversity induces tolerance, and when it may backfire. We discuss in how far the ingroup-projection model can integrate conflicting findings on the multiculturalism ideology.

## Introduction

How can intergroup bias be alleviated? Effective ways of reducing prejudice and improving intergroup relations are core themes of social psychology. A starting point of conflictual intergroup processes is a categorization into “us” versus “them” [1, 2]. Accordingly, much social-psychological research has developed models aiming to alter the level at which individuals categorize themselves and others, for example, the decategorization model [3] and the common ingroup-identity model (see [4]) (for a review, see [5]). Potential drawbacks related to each of these strategies have been identified, at the core of which is that individuals value their group identities [6] on an adequate level of abstraction [7]. The importance of (sub)group identities is also a core component of an ideology prominent both in northern America and western Europe, namely multiculturalism; it “explicitly acknowledges differences among groups and promotes the notion that differences associated with social identities should be valued and even celebrated” ([8], p. 85). Alas, whereas much empirical research has demonstrated positive outcomes of a multicultural orientation [9–12], this orientation also has negative consequences (e.g. [13, 14]). For example, highly identified White Americans reported increased prejudice after multiculturalism was activated [15].

Several researchers [16, 17] have pointed out similarities between the multiculturalism ideology and a recent model based on the social-identity perspective, the ingroup-projection model [18]. This model has been rigorously tested in different intergroup contexts, with many of its predictions confirmed (for reviews, see [19, 20]). According to the ingroup-projection model, a promising route to alleviate intergroup bias is defining the superordinate category, encompassing ingroup as well as outgroup(s), explicitly by its diversity. As the model assumes, with such a complex superordinate-category representation, the standards of the ingroup are no longer used to judge the outgroup, and intergroup bias is reduced. For example, if Canadians value their nation for being multicultural, White Canadians will not judge their non-White counterparts for behaving differently than them because they are seen as expressions of just that multicultural diversity. However, not all intergroup relations share the same properties. In particular, we argue that activating superordinate-group diversity could provoke more negative reactions among group members who consider their own group to represent the essence of the superordinate group (“pars pro toto” majorities, [21]), than among those who believe their group to be one among several (“una inter pares” minority groups). For example, similar to her Canadian counterpart, after thinking about the diversity of Europeans, a German who perceives her ingroup as just one among many in Europe may no longer use “German standards” to judge others, thus reacting tolerantly if Polish behave differently. In contrast, the same person, perceiving all Germans as being Caucasian like her, may, after thinking about the diversity of Germans, feel inclined to reinforce that being of Caucasian descent is essential to being German. In this case, perceived diversity on the superordinate category level may backfire on intergroup relations. The present research extends the ingroup-projection model by delineating under which conditions activating diversity as a feature of the superordinate category improves outgroup attitudes, and under which conditions it may impair them. We test this in three experiments. In the General Discussion, we discuss in how far the ingroup-projection model may provide a basis for multiculturalism research.

## Ingroup projection and intergroup bias

Particularly rich theoretical frameworks for specifying the conditions under which intergroup bias emerges are social-identity theory [1] and self-categorization theory [2]. Turner and colleagues [2] suggested that, based on perceived similarities and differences, individuals categorize themselves into groups they belong to (ingroups). In line with social-identity theory, such categorizations are assumed to be essential for intergroup bias comprising negative attitudes towards outgroups. Importantly, these models assume that similar psychological processes occur in many different intergroup contexts if people identify with the respective social groups. Within this research tradition, the ingroup-projection model, IPM [18], elaborates in particular how social groups are nested within one another and which implications this may have for intergroup bias. Before we turn to conditions that may alleviate intergroup bias, we need to review the model’s underpinnings that are relevant for our purposes.

The first assumption of the ingroup-projection model is that people apply the norms and standards of their ingroup to a superordinate category that includes outgroups along with the ingroup (‘ingroup projection’). For example, Germans may consider being hard-working a feature that all Europeans should possess. The resulting prototype is “the ideal-type member of a category that best represents its identity in a given context and frame of reference” on a superordinate level ([19], p. 335). A prototype is closely related to a stereotype, but varies with the context. In the present example, ‘the hard-working European’ would be the prototype. Due to ingroup projection, Germans, Italians, and Brits should all have a different perception of the prototypical European (see [22, 23], for evidence related to this example). The second assumption is that the higher people judge the typicality of their ingroup for the superordinate category (‘relative ingroup prototypicality’), the more outgroup bias they show. Findings supporting this assumption have been obtained in many different intergroup contexts (for recent findings, see [22, 24–27]); including those supporting a causal link [23, 28]. For example, the more psychology students thought their own group was more typical of students than business students, the more negative were their attitudes towards business students, and vice versa for business students [21]; for related findings, see [29]. Particularly, group members highly identified with both the ingroup and the superordinate category see the ingroup as relatively prototypical (for a review, see [19]).

## Superordinate-category complexity

The present research focuses on a third central prediction of the ingroup-projection model. Mummendey and Wenzel [18] proposed that a complex superordinate-category representation should increase tolerance and reduce intergroup bias. If the superordinate category is represented in a complex way, it is explicitly defined by its diversity [30]. Thus, diversity becomes a characteristic of the superordinate category, and outgroup attributes, though different from the ingroup’s, can be interpreted as similarly representative. For example, if people value their European identities precisely *because* of the differences between Brits, Germans, and Italians, these differences no longer lead to intergroup bias: Germans would regard both disciplined Brits and easy-going Italians as prototypical Europeans. This prediction of the ingroup-projection model may provide a theoretical foundation for prejudice-reduction strategies based on a multiculturalism ideology, acknowledging and appreciating diversity [16, 17]: “The most promising measure to reduce ingroup projection is in our view the establishment of a more complex representation of the superordinate category, made up of different prototypes, where a single group cannot reasonably claim to be the part that represents the whole” ([19], p. 366).

Testing this assumption, Waldzus and colleagues [31] manipulated the complexity of the superordinate-category representation of Europeans by asking participants to define the diversity versus the unity of Europe. Relative ingroup prototypicality was lower after diversity had been activated. Furthermore, as path analysis showed, a complex superordinate-category representation improved outgroup attitudes by reducing relative ingroup prototypicality (also see [23]). A similar effect of superordinate-category complexity on outgroup attitudes was also demonstrated in a real-life diversity training [32]. As a result of the diversity training, participants perceived the superordinate category *adults* as more diverse, which mediated the effect of the training on a sexism reduction. In sum, enhancing the complexity of superordinate-category representations via increasing perceived diversity can be a successful way to improve intergroup attitudes.

However, other research tried to extend these findings and yielded less encouraging findings [33]. In that research, participants were asked to think about one of two different intergroup contexts. In the first context, relying on the same manipulation as Waldzus and colleagues [23, 31], participants were asked to think about the unity versus diversity of the *world population* and then rate the prototypicality of members of *developed* and *developing countries* for the world population in general (following [24]). Replicating previous findings [23, 31] in a different intergroup context, typicality of industrial nations was rated lower and typicality of developing countries was rated higher in the diversity condition as compared with the unity condition. Also as predicted by the ingroup-projection model [34], in the diversity as compared with the unity condition, intentions to act against global inequality were higher. However, contradictory findings emerged in the intergroup context *students in general* (referring to secondary-education students in German), with *university students* and *polytechnic students* as subgroups. University students who had thought about diversity felt their group was *more* typical of students in general than those in the unity condition. Moreover, in the diversity as compared with the unity condition, attitudes towards the outgroup, polytechnic students, were more negative (rather than more positive). Thus, it appears that the positive effects of thinking about diversity do not generalize across all intergroup contexts.

## Perils of activating diversity

When theorizing about the potential perils of activating diversity, it is informative to consider the function of the ingroup prototype for group members. The superordinate category is an ingroup on a higher level of inclusiveness [2]. For example, both Germans and Europeans are ingroups for Germans. Therefore, if activating the diversity of the superordinate category changes the prototype representation, it may have negative consequences. For example, social-identity threat (for a review, see [35]) can emerge when the ingroup’s prototype is questioned and unclear in its definition [36–39]. Similarly, recent research has demonstrated perils of a multiculturalism ideology [40, 41]: It fails under conditions of high conflict [16]; creates a preference for individuals who remain within the limits of their own ethnic group [13]; dominant group members may feel excluded by this perspective [14]; and priming multiculturalism may lead to higher prejudice among highly-identified majority members ([15], but see [42]).

These findings indicate that activating diversity of a superordinate category does not necessarily improve intergroup relations. Thus, the crucial question is: Under which conditions does activating diversity improve intergroup relations, and when does it impair them? The present research focuses on one feature of the intergroup setting: whether ingroup and superordinate category are highly related, such that the ingroup is perceived to essentially represent the superordinate category, or not. Henceforth, we refer to such ingroups as majority (versus minority) ingroups. Within the ingroup-projection literature, it has been proposed that “developing a more complex representation of the superordinate category implies a more equal status of the included groups; the majority will probably perceive this as a relative status loss, whereas the minority should see it as a status gain. Thus, the complexity of the superordinate category could itself become an issue of contention and conflict” ([19], p. 364). In line with those authors’ suspicion, we assume that for majority group members who perceive their ingroup as highly prototypical for the superordinate category, activating diversity may question their group’s position in the superordinate category and thus elicit threat and negative outgroup attitudes (for similar ideas, see [43]). An example of such a majority group in the research cited above are university students as prototypical secondary-education students [33]. In contrast, for minority-group members who perceive their ingroup as low in prototypicality, activating diversity should draw attention to other groups included in the superordinate category, thus provoking a complex representation, reducing ingroup projection and improving outgroup attitudes. Example ingroups investigated in previous research are Germans as Europeans [23, 31] and members of industrial societies as members of the world population [33].

In a nutshell, the ingroup-projection model originally assumes that a diversity manipulation should *reduce* ingroup projection (i.e., reduce the tendency to think that the superordinate category resembles the ingroup, but see [44]). We propose instead that ingroup prototypicality is not only an outcome variable, but can also be an important moderator. We assume that in majority-ingroup members, a diversity manipulation can also induce threat.

## Overview of the present research

The aim of the present research was to test whether activating diversity can provoke opposite psychological processes as a function of perceived ingroup prototypicality (Hypothesis 1). We hypothesized that activating diversity should be threatening (Hypothesis 2) and thus impair outgroup attitudes (Hypothesis 3) among those majority-group members who see their ingroup as highly prototypical for the superordinate category. For those who see their ingroup as less prototypical, previous findings on ingroup projection should be replicated: Activating diversity should improve outgroup attitudes (Hypothesis 4). The main aim of Experiment 1 was to test the hypothesis that activating diversity can be threatening for those participants who see their ingroup as high in relative prototypicality, but not for those who see their ingroup as less prototypical. Only a preliminary test was undertaken whether this threat also affects outgroup attitudes. Experiment 2 also tested the interaction of activating diversity and perceived ingroup prototypicality, but other than in Experiment 1, the focus was on outgroup attitudes. Experiment 3 then manipulated prototypicality in order to provide causal support for its crucial role in eliciting threat (i.e., for Hypothesis 1).

## Experiment 1

The main aim of Experiment 1 was to test whether activating superordinate-category diversity can lead to threat in those majority members who see their ingroup as relatively high in prototypicality. We selected *heterosexual men* within the superordinate category *men in general* as the majority in question. A reason for this choice was that male identity is characterized by a precariousness that seems to require frequent validation because it is elusive (for a review, see [45]). When this identity is questioned, threat leads to behavior that aims at restoring the threatened identity [46–48]. Identity threat, in turn, relates to intergroup bias (for a meta-analysis, see [49]). Whereas scales exist that measure different aspects of threat, the precise construct we were interested in was threat to the male prototype. We therefore constructed a scale intended to measure prototype threat, with some items being formulated in general terms and others referring to threat imposed by other groups.

With our main focus on threat, we used a one-item affective measure (cf. [50]) as a preliminary test of the relationship with outgroup attitudes. A final, secondary aim of the present study was to distinguish the role of relative prototypicality from that of identification: In the context of multiculturalism research, ingroup identification has been identified as a crucial moderator variable for majority members’ intergroup attitudes [15]. By additionally measuring identification, we tried to exclude the alternative explanation of our findings that identification is the crucial moderator that is highly correlated with relative prototypicality.

We expected an interaction of relative prototypicality and experimental condition on threat. We hypothesized that those participants who perceive their ingroup as highly prototypical for the superordinate category should perceive more threat in the diversity as compared to the unity condition. In contrast, for participants who perceive their ingroup as relatively low in prototypicality, we expected that activating diversity should not provoke threat, as compared with the unity condition. Additionally, a preliminary test of moderated mediation was conducted: Because of perceived threat, participants who perceive their ingroup as highly prototypical should report more negative outgroup attitudes in the diversity as compared with the unity condition.This should not be the case for participants who perceive their ingroup as relatively low in prototypicality.

### Method

#### Ethics statement

Approval to conduct the present experiment was given by the Ethics Commission of the Faculty of Psychology of the University of Koblenz-Landau (2015–48).

#### Participants and design

Participants were 100 male students (*M*_age_ = 23, *SD* = 3) from a polytechnic university in Germany. They were recruited on campus and volunteered to participate for a reward of 2€. Given that the criterion for ingroup membership was heterosexual orientation, two participants were excluded from the analyses. Participants were randomly assigned to either the diversity or the unity condition. Sample size was determined using an a-priori power analysis. Given one between-subjects factor with two levels (experimental condition: unity vs. diversity) and relative prototypicality as continuous moderator, *N* = 98, and α = .05, a medium-sized regression effect of *R*^*2*^ = .15 [51] could be detected in a two-tailed test with a statistical power of 1 –β = .95 [52].

#### Materials and procedure

Participants took part in “three unrelated studies” at separate tables in a large room at the polytechnic. After they had given written informed consent and completed a short filler task, the experimenter handed them the questionnaire that contained the diversity manipulation. Then, they received an allegedly unrelated questionnaire including the threat measure, the measures of prototypicality, the outgroup attitude measure, identification, and demographic variables (including sexual orientation). At the end of the experiment, participants were thanked, paid, and debriefed.

Manipulation of superordinate-category diversity: We aimed at increasing perceived variability within the group by drawing attention towards differences among group members or subgroups [53]. Covered as a pretest for a future study on “the dynamic male image”, we asked participants in the diversity condition to think about as many groups as possible that can be part of the superordinate category men, and write them down (cf. [54]). In the unity condition, we asked participants to think about “traditional manhood”. Analogous to the diversity condition, we asked participants to think about as many groups as possible, and write them down. Thus, participants in the control group were also thinking about men, yet not about their diversity.

Measures of prototypicality: Prototypicality of both groups was measured indirectly, as has been established in previous studies (e.g. [21, 31]). Attributes were generated in a separate pretest with *N* = 36 male students who were asked to generate and name attributes that are typical of gay versus heterosexual men. Based on the frequency of attributes mentioned, four attributes were selected that were seen as typical of gay men (German: gepflegt, sensibel, modebewusst, feminin; translations: neat, sensitive, well-dressed, feminine) and four that were typical of heterosexual men (German: stark, sportbegeistert, technikbegabt, maskulin; translations: strong, keen on sports, talented for technology, masculine). Participants in the experiment proper were asked to rate these attributes in terms of their applicability to *gay men*, *heterosexual men*, and *men in general*, with the order of the rating for the two subgroups counterbalanced and the superordinate category always rated last. Responses were given on 7-point Likert-type scales (from 1, “I absolutely agree” to 7, “I absolutely disagree”) and the Euclidean distance [31, 55] between correspondent trait ratings served as the indicator of the typicality of the respective subgroup for the superordinate category. In detail, the differences between the ratings for the subgroup and superordinate category were squared, summed, and the square root was taken. Then, scores were recoded so that larger scores (more intuitively) reflect higher prototypicality. Ingroup prototypicality minus outgroup prototypicality yielded the index of relative prototypicality, with higher values indicating higher relative ingroup prototypicality.

There is an alternative way to analyze prototypicality ratings: To use the prototypicality ratings of both subgroups separately, instead of a relative measure, to predict outcomes. Separate analyses of ingroup and outgroup prototypicality are presented in Appendix 1.

Measure of perceived threat: We formulated eleven statements intended to measure perceived threat to the male prototype, again with a seven-point Likert-type scale. About half of the items were formulated in general terms (e.g., “Many others tell us how we as men ought to behave.”, “The influence of the media with regard to the male image hinders me to feel as I want to as a man.”), the others referred to the threat imposed by other groups (i.e., women and other men, e.g., “As a man, I feel uncomfortable when I think about how some men influence the male image by their out-acted sexuality.”, “I don’t care about the thought that women might find gay men attractive.”^R^). To explore the internal structure of this yet unvalidated measure, a principal components factor analysis with Oblimin rotation was computed after data collection. Two strong factors emerged (Eigenvalues: 3.88 and 1.59, variance explained: 35% and 14%, respectively), reflecting *general threat* and *other-group threat*. All other factors were considerably weaker (Eigenvalues < 1.01), and an inspection of the scree plot confirmed the decision to extract two factors. Six items loaded mainly on the first factor (“general threat”, α = .80), five others loaded mainly on the second factor (“other-group threat”, α = .63), see Appendix 2.

Outgroup attitude: A one-item affective measure was taken from an established scale [56] and used to assess the negativity of the outgroup attitude (“I think gay men are repulsive.”, scale: 1–7). It was log-transformed to approach a normal distribution because attitudes towards gay men were skewed towards the tolerant end of the scale, a typical finding with German students [57, 58].

Identification: We used five items to measure identification with the ingroup of heterosexual men and five to measure identification with the superordinate group of men in general (e.g., “I feel strongly related with other men.”, “I am glad to be a heterosexual man.”, scale: 1–7). However, a factor analysis demonstrated that a one-factor solution was more adequate, indicating that heterosexual men perceive a huge overlap between heterosexual men and men in general; thus, we formed a 10-item identification scale (α = .78).

### Results

In all analyses in the present article, the type I error level was set at α = .05. As an indicator of the effect size, *R*^*2*^_*p*_ is reported [51] which is numerically identical to partial η^2^. Throughout, estimated marginal means are reported. The raw data of all reported experiments can be found as supporting information (S1 File, S2 File, and S3 File for Experiments 1, 2, and 3, respectively).

#### Relative prototypicality

We first tested whether relative prototypicality was unaffected by the manipulation, as we had assumed, conducting a 2 (experimental condition) × 2 (perceived prototypicality of gay vs. heterosexual men) ANOVA with repeated measures on the second factor. There were no main or interaction effects involving the manipulation on the perceived prototypicality of heterosexual men or that of gay men, all *F*s < 1. In other words, experimental condition was uncorrelated with perceived prototypicality of each subgroup and uncorrelated with relative prototypicality, all *|r|* < .06. We thus used prototypicality as a moderator variable. In addition, as one would assume, heterosexual men perceived their ingroup as closer to the prototype of *men in general* than they perceived gay men (*M* = 7.82, *SEM* = .14 vs. *M* = 5.08, *SEM* = .25), *F*(1,95) = 130.58, *p* < .001, *R*^*2*^_*p*_ = .58.

#### Moderation of the effect of diversity activation by relative prototypicality

We hypothesized that threat would result as a function of experimental condition and relative prototypicality. In order to test this hypothesis, we coded the experimental factor with –1 for the unity condition and 1 for the diversity condition and computed an interaction term between condition and centered relative-prototypicality scores. Experimental condition and relative prototypicality were entered simultaneously as predictors of general threat and other group threat, respectively, in multiple regression analyses. No statistically significant interaction was found on *general threat*, *B* = .10, *SE* = .06, *β* = .17, *t* = 1.69, *p* = .10, nor was the regression model significant (*F* < 2.01, *p* = .12; for the model without the interaction: *F* < 1.55).

**Fig 1:**
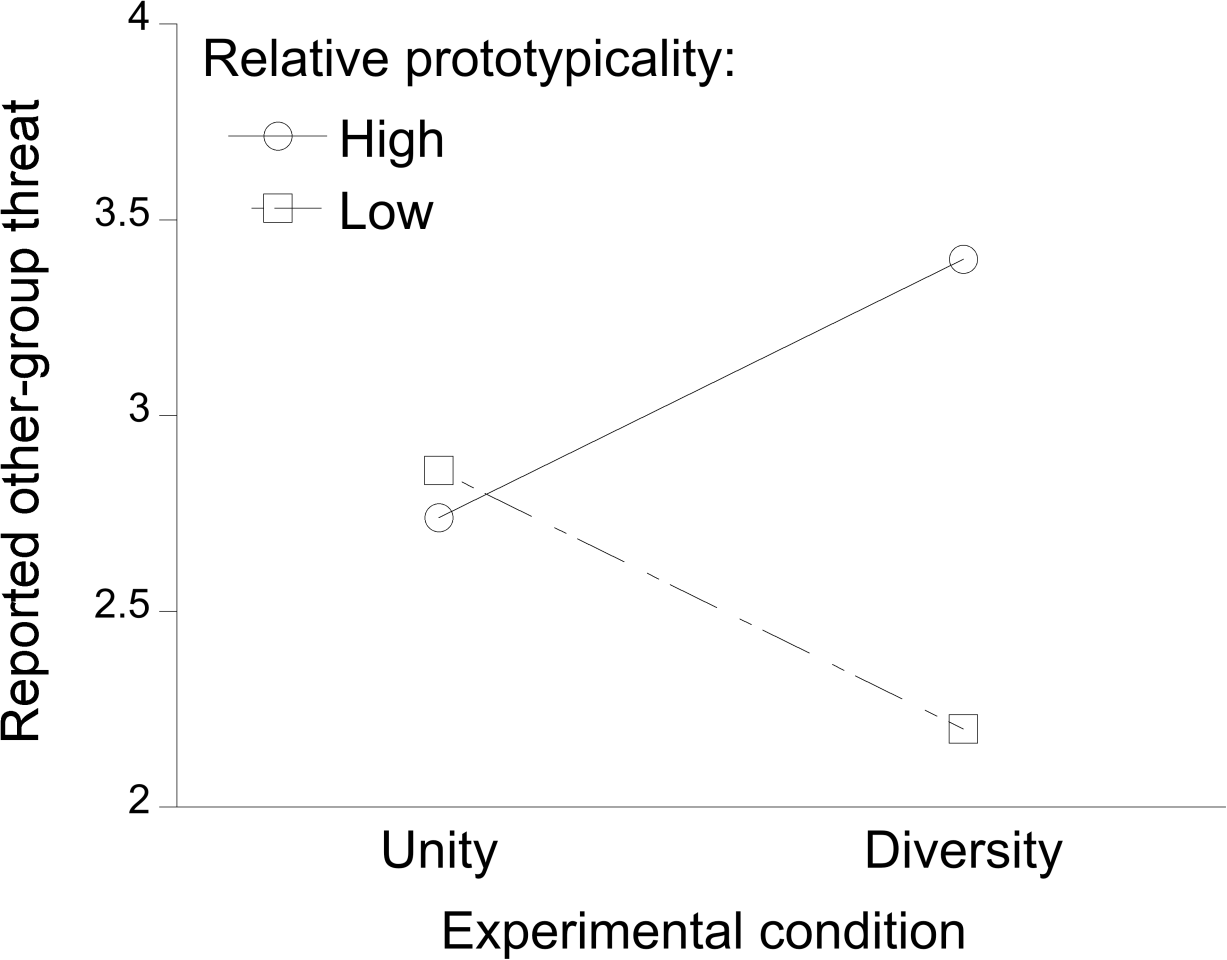
Experiment 1: The effect of the experimental condition (activating unity vs. diversity of the superordinate category *men*) on perceived threat, for participants who perceived the ingroup heterosexual men as much more prototypical (relative prototypicality: high) than gay men, or not (relative prototypicality: low).

For *other-group threat* as the dependent variable, the overall regression model without the interaction resulted in *R*^*2*^ = .06, *F*(2,94) = 2.77, *p* < .07. Experimental condition did not affect threat, *β* = .00, whereas participants who perceived their ingroup to be relatively more prototypical for the superordinate category reported significantly more threat than participants perceiving lower prototypicality, *B* = .11, *SE* = .05, *β* = .24, *t* = 2.36, *p* = .02. More central to our hypothesis, with the interaction term entered in the second step of the regression, there was a significant increase in explained variance in other-group threat, Δ*R*^*2*^ = .10, Δ*F*(1,93) = 10.36, *p* = .002 (confirming H1, see Fig 1). Thus, relative prototypicality was a significant moderator of the relationship between experimental condition and other-group threat, *B* = .14, *SE* = .04, *β* = .31, *t* = 3.22, *p* = .002.

To assess the nature of the interaction, simple slopes were computed. Among participants perceiving their ingroup to be relatively low in prototypicality, the slope was *B* = –.33, *SE* = .15, *β* = -.31, *t* = -2.27, *p* = .025. This indicates that participants who perceived low relative prototypicality of their ingroup experienced less other-group threat in the diversity compared with the unity condition. In line with H2, this effect was reversed among participants perceiving their ingroup to be highly prototypical for the superordinate category, *B* = .33, *SE* = .15, *β* = .31, *t* = 2.29, *p* = .02, indicating that they perceived significantly more other-group threat in the diversity compared with the unity condition. Conversely, the slope in the diversity condition between low (1 *SD* below the mean) and high (1 *SD* above the mean) relative prototypicality was *B* = .26, *SE* = .06, *β* = .56, *t* = 4.03, *p* < .001, indicating that participants perceiving their ingroup to be more prototypical for the superordinate category experienced more threat than participants perceiving lower prototypicality. In the unity condition, the degree of relative prototypicality had no impact on perceived threat, *β* = -.06, *|t|* < 1.

#### Outgroup attitudes and moderated mediation

We next conducted a preliminary test of whether there was an effect of the experimental condition on the attitude item, mediated by threat, and whether this mediation was moderated by perceived prototypicality (following [59], Model 7). We allowed the effect of experimental condition on the mediator other-group threat to be moderated by perceived prototypicality. Replicating what we reported above, there was no main effect of experimental condition on perceived threat, but both an effect of perceived prototypicality and an interaction between both variables. The regression analysis in which outgroup attitudes were regressed on experimental condition and threat yielded only a significant effect of threat, *B* = 0.23, *SE* = 0.06, *t* = 3.92, *p* < .001 (experimental condition, *|t|* < 1). A bootstrapping analysis with bias-corrected confidence intervals and 10,000 bootstrap re-samples confirmed that there was no direct effect of experimental condition on outgroup attitudes (*B* = –0.06, *SE* = 0.06, *|t|* < 1). For participants who perceived the ingroup as high in relative prototypicality (1 *SD* above the mean), thinking about diversity induced threat and thus led to more negative outgroup attitudes (i.e., indirect effect of experimental condition via threat on outgroup attitudes, confirming H3: *B* = 0.08, *SE* = 0.04; 95% *CI*: 0.01; 0.16). For participants who perceived the ingroup as relatively low in prototypicality (–1 *SD*), thinking about diversity reduced threat and thus made outgroup attitudes more positive (H4, *B* = -0.07, *SE* = 0.04; 95% *CI*: –0.16; –0.01). No mediation was found at the mean of the moderator variable. In sum, these findings are in line with the following idea: Participants who perceive heterosexual men as much more typical men than gay men are affected by our activation of diversity in that they experience more threat to the male prototype than those in the control condition, and this high level of threat translates into more negative attitudes towards the outgroup. In contrast, and somewhat unexpected, participants who perceive heterosexual men as only a bit more typical than gay men are affected by our activation of diversity in that they experience less threat than those in the control condition, and this reduced level of threat improves attitudes towards the outgroup.

*Identification*. A similar analysis as above with identification as a potential moderator variable showed that heterosexual men’s higher ingroup identification went along with more negative outgroup attitudes, *B* = .14, *SE* = .07, β = .21, *p* = .04, replicating previous studies. However, identification did not moderate the relation between experimental condition and outgroup attitude (β = .01, *|t|* < 1). Moreover, identification was not a significant predictor of threat (β = .08, *|t|* < 1), nor did it moderate the effect of experimental condition on threat (β = .02, *|t|* < 1). Identification did not significantly correlate with perceived prototypicality of gay men, heterosexual men, or perceived relative prototypicality (|*r*s| < .12, *p* > .26). Therefore, we are positive that identification is not the moderator of the effect of diversity activation in the present study.

### Discussion

The findings of Experiment 1 largely corresponded to our hypotheses. Prototypicality appeared to be unaffected by the manipulation: Not surprisingly, even after thinking about the diversity of men, participants still thought that heterosexual men were considerably more typical men than gay men. An implication for ingroup-projection research is that this manipulation seems ineffective for large, highly prototypical majorities. Importantly, the effect of the manipulation was qualified by perceived relative prototypicality as expected. Those who perceived heterosexual men to be much more typical than gay men for men in general perceived more threat to the group prototype when the diversity of men was activated, and, in turn, reported more negative outgroup attitudes. This is fully in line with the notion [19] that a more diverse representation of the superordinate category may imply a relative status loss for the majority (also see [43]). Among participants who perceived gay men to be relatively high in prototypicality, the activation of diversity did not leave threat unaffected, as we hypothesized, but even decreased threat and thus improved outgroup attitudes compared to the unity condition. For them, in line with the ingroup-projection model, activating diversity probably yielded a complex representation that positively affected intergroup bias. Thus, Experiment 1 demonstrated that such positive effects are restricted to a subgroup of participants.

In the present study, ingroup projection was unaffected by activating diversity, even though diversity activation provoked threat. In contrast, previous research indicates that a threat manipulation may *increase* ingroup projection [44]. We assume that this difference in findings is due to the large majority group we chose (i.e., heterosexual men) who remain highly prototypical even in the light of group diversity: Being heterosexual still appears prototypical of men, even considering their diversity. In such cases, we assume that relative ingroup prototypicality is unaffected by a diversity manipulation. Still, from a methodological point of view, it would be desirable to measure relative prototypicality after the manipulation. Therefore, we adapted this procedural weakness of Experiment 1 in Experiments 2–3.

The manipulation we chose in Experiment 1 to create superordinate group complexity differed from that used in previous research. Whereas Waldzus and colleagues [31] had asked participants to think about the diversity or unity of the superordinate group, we asked participants to list different subgroups of men, either introduced as “the dynamic male image” or “traditional manhood”. One could argue that the different manipulations are the reason for the different patterns of findings. Therefore, in Experiments 2–3, our manipulations were more similar to those used in previous research.

The moderated mediation analysis yielded findings in line with our hypothesis regarding negative outgroup attitudes. Yet, we measured attitudes rather crudely in Experiment 1, using a one-item measure. Additionally, we wanted to exclude the possibility that carry-over effects occurred, given the preceding threat measure. Therefore, stronger support for the effects on outgroup attitudes was sought in Experiment 2 by omitting the threat measure.

## Experiment 2

The aim of Experiment 2 was to extend the findings of Experiment 1 by testing direct effects of inducing diversity on outgroup attitudes. Also, we improved the procedure by measuring relative prototypicality before the manipulation. Similar to Experiment 1, the context was heterosexual adolescents’ attitudes towards same-gender lesbian or gay peers. Only for those participants who perceived low relative ingroup prototypicality, we expected to replicate the finding [23, 31] that attitudes are more positive when superordinate-category diversity is activated (again called H4). In contrast, for those participants who perceived the ingroup as relatively high in prototypicality for the superordinate category, we expected more negative attitudes towards lesbians or gay men in the diversity as compared to the unity condition (H3), so there should be an interaction of condition and perceived relative prototypicality (H1).

### Method

#### Ethics statement

Approval to conduct the experiment was granted by the Ethics Commission of the Faculty of Psychology, University of Amsterdam (2016-SP-6516). The present research was carried out at school during breaks. In addition to participants, the school principal and parents gave their written informed consent.

#### Participants and design

Participants were 81 students of an upper-track high school (“Gymnasium”) in Germany. They were invited to take part in a study on adolescents’ conceptions of masculinity and femininity. Sexual orientation was measured by asking to which gender participants felt sexually attracted. Participants were excluded from analyses if they reported predominantly same-gender attraction. Additionally, participants whose written responses clearly indicated that the manipulation had activated thoughts in the opposite direction than intended were excluded, leaving a total *N* = 77 (*M*_age_ = 17.14 years, *SD* = 1.07, 45 girls, 32 boys). The pattern of findings reported is identical if all participants are retained. Participants had the chance to take part in a lottery to win vouchers for an online shop worth 10 € each.

The design was identical to that of Experiment 1, with one between-subjects factor with two levels (experimental condition: unity vs. diversity) and relative prototypicality used as a continuous moderator. Participant gender influences attitudes towards lesbians and gay men (e.g. [60]). Therefore, gender was included either as a factor or as a covariate in the analyses below, with linear relationships between covariates and dependent variables as well as homogeneity of regressions tested and confirmed. Main dependent variables were attitudes towards same-gender lesbian or gay peers.

#### Materials

Manipulation of superordinate-category diversity: The manipulation was closely modeled after [31]. Participants read the instruction to “describe girls [boys] in general, in all their diversity” versus “describe how typical girls [boys] are”. This instruction was presented on a sheet of paper that showed several pre-tested pictures of same-gender peers who were either highly prototypical (e.g., girls with flowers or mirrors) or diverse (e.g., girls playing soccer or as construction workers), depending on the experimental condition. They were asked to write down their thoughts about the diversity versus the typicality of their gender group in the space on the lower half of the page. The manipulation check on the next page consisted of the statement “Girls [boys] are…” with a scale from (1) “all very different” to (7) “all very similar”.

Measure of relative prototypicality: For measuring prototypicality, as several previous studies, we used direct rating scales [26, 54]. Participants responded to two statements, on scales from 1–7 (anchored: “I don’t agree at all” to “I absolutely agree”): “In my opinion, lesbian girls (heterosexual girls) [gay boys (heterosexual boys)] are typical girls [boys].” Again, we computed relative prototypicality as the difference between the two ratings. Separate analyses for ingroup and outgroup prototypicality are reported in the Appendix.

Attitude measures: Given that our theoretical predictions concern cognitive representations, we measured the cognitive component of attitudes towards lesbian girls and gay boys. Analyses are based on three averaged age-adequate items (“At school, more should be done for lesbian [gay] students^R^.”, “A lesbian [gay] school captain would be detrimental to our school’s reputation.”, “Lesbian girls [gay boys] should better not talk about their sexual orientation.”, scale: 1–7, α = .78), coded such that larger scores represent more negative attitudes.

#### Procedure

All participants started by giving their informed consent to respond to a questionnaire on masculinity and femininity. Then, 16 warm-up questions were presented about the attributes ascribed to girls (for girls) and boys (for boys) taken from the Personal Attributes Questionnaire [61]. Afterwards, the prototypicality of heterosexual and lesbian girls [gay boys] was assessed, followed by the manipulation, a manipulation check, and the attitude questionnaire. The study ended with demographic questions and a thorough written debriefing.

### Results

#### Manipulation check

Girls and boys reported that members of their gender group were somewhat less similar to each other in the diversity than unity condition (*M* = 2.66, *SEM* = .22, vs. *M* = 2.89, *SEM* = .21), but this descriptive difference was not statistically significant, *F* < 1, possibly due to the high proportion of unexplained variance.

#### Prototypicality

As relative prototypicality was assessed before the manipulation, it should be unaffected by it, unless a randomization error occurred. Indeed, a 2 (experimental condition) × 2 (participant gender) × 2 (target sexual orientation) ANOVA with repeated measures on the last factor showed that there were no main effects or interactions on prototypicality measures involving the manipulation, all *F*s < 1.

Again, unimportantly, heterosexual girls and boys (*M*s = 5.20 and 5.58, *SEM*s = 0.24 and 0.29) were perceived as more typical for their gender group than lesbian girls or gay boys (*M*s = 3.46 and 2.41, *SEM*s = .21 and .25), *F*(1,72) = 80.54, *p* < .001, *R*^*2*^_*p*_ = .53. This difference was larger for boys than girls, interaction: *F*(1,72) = 6.87, *p* = .01, *R*^*2*^_*p*_ = .09 (gender main effect: *F* < 2.22). The extent to which heterosexual boys were perceived to be more typical boys than gay boys was larger than the extent to which heterosexual girls were perceived to be more typical girls than lesbian girls, simple main effects: *F*(1,72) = 56.60, *p* < .001, *R*^*2*^_*p*_ = .44, and *F*(1,72) = 24.60, *p* < .001, *R*^*2*^_*p*_ = .26, respectively. Gay boys were perceived to be less typical of boys than lesbian girls were perceived to be of girls, *F*(1,72) = 10.23, *p* < .01, *R*^*2*^_*p*_ = .12. In contrast, heterosexual boys were not perceived to be significantly more typical of boys than heterosexual girls were perceived to be of girls, *F* < 1.

#### Attitudes

A 2 (experimental condition) × 2 (gender) ANOVA yielded no hint that average attitudes were more positive after activating diversity rather than unity. The only effect we found was that girls’ attitudes towards lesbian peers were less negative than boys’ attitudes towards gay male peers, *F*(1,72) = 12.37, *p* = .001, *R*^*2*^ = .15 (*M*s = 3.14 vs. 4.30, *SEM*s = .21 and .25), respectively, both other *F*s < 1). In other words, thinking about diversity did not generally lead to more positive attitudes than thinking about unity.

#### Moderation of the effect of diversity activation by relative prototypicality

**Fig 2:**
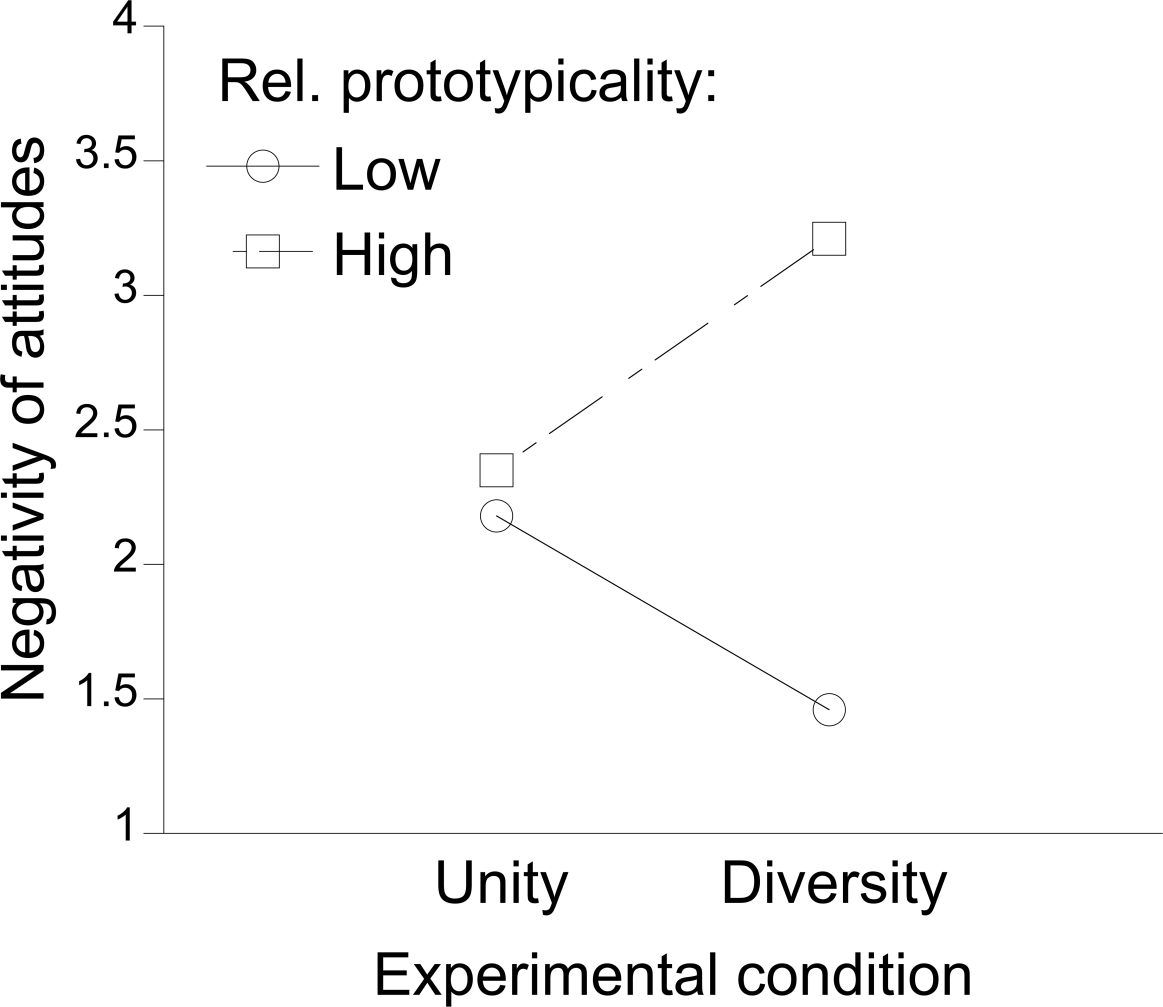
Experiment 2: The effect of the experimental condition (activating diversity vs. unity of the gender group) on attitudes towards lesbian girls or gay boys, respectively, for participants who perceived the ingroup (heterosexual girls/boys) as high versus low in relative prototypicality as compared to the outgroup (lesbian girls/gay boys).

In order to test H1 that perceived relative prototypicality moderates the effect of the experimental condition, we again used a regression-based approach [62]. Controlling for participant gender, we tested whether attitudes towards lesbian girls or gay boys, respectively, depended on experimental condition (–1: unity; 1: diversity) and centered relative prototypicality scores. The regression model was statistically significant, *F*(3,71) = 8.70, *p* < .001, *R*^*2*^ = .27. Higher relative prototypicality was related to more negative attitudes, *B* = .23, *SD* = .07, β = .37, *t* = 3.44, *p* = .001, and boys’ attitudes towards gay boys were more negative than girls’ attitudes towards lesbian girls, *B* = .85, *SD* = .32, β = .28, *t* = 2.64, *p* = .01, but there was no main effect of experimental condition, *|t|* < 1 (see ANOVA above). Most importantly, in Step 2, we found the expected interaction of experimental condition and relative prototypicality (confirming H1), *B* = .16, *SD* = .06, β = .26, *t* = 2.60, *p* = .01. Including the interaction significantly increased the explained variance in attitudes, Δ*F*(1,70) = 6.75, *p* = .01, Δ*R*^*2*^ = .06 (see Fig 2). Simple slopes analyses showed that given perceived low relative prototypicality of heterosexual girls or boys, attitudes tended to be more positive in the diversity than unity condition. This finding conceptually replicates Waldzus and colleagues’ finding [31], *b* = –.33, *SD* = .21, β = –.22, *t* = –1.62, *p* = .11, but the effect was not statistically significant, yielding only weak support for H4. In contrast, given perceived high relative prototypicality of heterosexual girls or boys (1 *SD* above the mean), in line with H3, attitudes were significantly more negative in the diversity than unity condition, *b* = .45, *SD* = .21, β = .30, *t* = 2.11, *p* < .04.

All of these findings were replicated in an additional unpublished experiment (*N* = 143) in which prototypicality was not assessed at the beginning as we did here, but towards the end of the questionnaire (as is usual practice in research on ingroup projection and as we did in Experiment 1) [63].

The inclusion of the Personal Attributes Questionnaire at the beginning allowed us to test an alternative hypothesis. Gender belief systems have been identified as an important factor explaining attitudes towards lesbians and gay men [60, 64]. One might suspect that indicating how prototypical heterosexual girls/boys and lesbian girls /gay boys are for their gender group in general is nothing but an alternative operationalization of this known construct. However, gender role endorsement did not moderate findings (all |β|s < .12, *p*s > .24).

### Discussion

In the present experiment, after activating a diverse compared to a homogeneous representation, we did not find generally more positive outgroup attitudes. Instead, perceived relative prototypicality moderated the effect of activating diversity on outgroup attitudes. Given perceived low relative prototypicality of heterosexual as compared to lesbian girls or gay boys, we replicated the ingroup projection finding that attitudes are more positive in the diversity than unity condition, but only descriptively. In contrast, for participants who perceived lesbian girls or gay boys as much lower in prototypicality than heterosexuals, attitudes were more negative in the diversity than unity condition.

Experiment 2 used a different manipulation of diversity than Experiment 1 and yielded a similar interaction pattern on outgroup attitudes as Experiment 1 had shown on threat. It thus appears that comparable effects result from the different manipulations.

In short, our findings demonstrate that activating diversity is not beneficial to counteract intergroup bias among all participants. However, our proposed moderator variable, prototypicality, was again measured, not manipulated. What is still missing is causal support on its role. Additionally, it was desirable to investigate the hypothesized processes in a different intergroup context.

## Experiment 3

The aims of Experiment 3 were to manipulate prototypicality perceptions and to test whether activating diversity has positive effects when ingroup prototypicality is relatively low (and outgroup prototypicality, high), but provokes threat when ingroup prototypicality is relatively high (and outgroup prototypicality, low). Previous research reported difficulties to successfully manipulate prototypicality perceptions even in artificial groups [34]. Probably, it is threatening per se to inform members of highly prototypical groups that their group is not so prototypical after all. For example, telling university students that polytechnic students are higher in number and status than their own group may pose an ingroup threat. As a case in point, White Americans felt threatened when reminded of their future minority status in the U.S. [43]. In light of these findings, we opted for an alternative manipulation: We manipulated the intergroup context while holding constant the ingroup (similar to [22, 23]). Our participants were members of an ingroup (U.S. Americans) that, as we assumed, is perceived as high in prototypicality in one superordinate category context (i.e., North America) where it represents a majority. In a different intergroup context (i.e., the world population), U.S. Americans should perceive their ingroup as lower in prototypicality. Likewise, we expected that the outgroups, non-U.S. Americans, are regarded low in prototypicality in the context of North America, but highly prototypical in the context of the world population. We asked U.S. Americans to rate the prototypicality of in- and outgroups in either one or the other of these contexts. Then, as in the previous experiments, they either thought about the unity or about the diversity of the respective superordinate category, followed by a measure of threat focusing on leadership entitlement of the ingroup in the political context.

Our hypotheses were as follows. First, if the manipulation of the superordinate category worked as intended, participants should regard U.S. Americans as more prototypical than the outgroups in the superordinate category *North Americans;* in contrast, in the superordinate category *world population*, the outgroups should appear more prototypical than U.S. Americans. More substantially, as a consequence, we should find an interaction of both experimental factors, prototypicality in the given context (North America vs. world) × diversity versus unity (H1). Thinking about diversity should induce threat (H2) in the context of the superordinate category *North Americans* as compared to the context *world population*, and this perceived threat should lead to more negative outgroup attitudes (H3). No such negative effects of context were expected in the unity conditions.

### Method

#### Ethics statement

The study was approved by the Ethics Commission (Faculty of Psychology, University of Amsterdam, 2015-SP-6337), and informed consent by the adult participants was collected. Only if they clicked the button that they agreed to participate in the study, could they proceed.

#### Participants and design

In order to detect a medium-size interaction effect (*f* = .25) in a 2×2 ANOVA with a statistical power of 1 –β = .95, 210 participants were needed. We were able to recruit 200 adults online via MTurk who were compensated with $1 for participating in an English-language study on political issues concerning the U.S. They were randomly assigned to four experimental conditions. We excluded those from analyses who indicated they were not U.S. Americans (*n* = 12). Additionally, as in the previous experiment, we excluded those whose responses indicated the contrary of the intended manipulation (*n* = 17, e.g., “There are no typical North Americans” in the unity condition; “I don’t think there are many differences at the core” in the diversity condition), leaving 41 participants in the US, unity, 44 in the world, unity, and 43 in the US and world diversity conditions, respectively. Whether the latter participants were excluded or retained did not affect the pattern of results. A post-hoc power analysis showed that given *N* = 171, the actual statistical power was 1 –β = .90.

Of these participants, 42% indicated to be female and 58% to be male; they ranged in age from 18 to 67 years (*M* = 34.57, *SD* = 11.86). Based on their self-categorizations, we classified 72% as Caucasians, 9% as African Americans, 8% as Asian, 2% as Hispanic, and 9% as mixed/other. Whereas 50% identified as atheists, 50% indicated various religious faiths. Religiosity has proven to be an important variable in different intergroup settings (e.g. [48, 65]). Because it had substantial effects on the dependent variables, whether people were atheists or not was included as a covariate in the analyses below.

The design was a 2 (experimental condition: unity vs. diversity) × 2 (context: North America vs. world) between-subjects design. Main dependent variables were perceived threat and outgroup attitudes.

#### Materials and procedure

Manipulation of prototypicality and manipulation check: After giving informed consent, participants were asked to take a moment to think about the role of “U.S. Americans in the context of North America” or think about the role of “U.S. Americans in the context of the world in general”. This was immediately followed by the manipulation check: measures of prototypicality that depended on the prototypicality condition. Participants responded to two statements, on continuous scales from 0–100 (“do not agree at all” to “completely agree”): “In my opinion, U.S. Americans are THE typical people in North America [in the world]” and “In my opinion, those who are not U.S. Americans are THE typical people in North America [in the world]”. As ingroup and outgroup prototypicality were manipulated at once in this experiment, no differentiated analyses are presented in the Appendix.

Manipulation of superordinate-category diversity: After the manipulation check, we asked participants to think about the diversity [unity] of people in North America [in the world in general], depending on the experimental condition (unity vs. diversity) and on the prototypicality condition (high vs. low). They were asked to type their thoughts into the space provided.

Threat measure: Next, we measured threat to the ingroup’s leadership entitlement, on a 1–7 scale (anchored “do not agree” to “agree completely”), using the item “The U.S. should have the most important voice in international politics”.

Attitude measures: Given that different intergroup contexts were activated, we assessed attitudes towards “people from other countries” with four items (e.g., “I like to meet people from other countries, in general.”; “When I think of people from other countries, I have positive feelings.”; α = .93).

Demographic information: Finally, demographic information was collected (age, gender, racial identification, nationality, religious denomination). Afterwards, participants were debriefed with a short summary of the study aims.

### Results

#### Manipulation check

As expected, U.S. Americans considered their ingroup as prototypical and the outgroups as non-prototypical in the context of North America (*Ms* = 71 vs. 33, *SEM*s = 2.63 and 2.73, respectively), but their ingroup as non-prototypical and the outgroups as prototypical in the context of the world (*Ms* = 35 vs. 57, *SEM*s = 2.58 and 2.68, respectively). This finding was confirmed in a 2 (context condition) × 2 (ingroup vs. outgroup) ANOVA with repeated measures on the second factor, *F*(1,164) = 83.52, *p* < .001, *R*^*2*^_*p*_ = .34. Of minor interest, as the means above indicate, there was also a main effect of context condition, *F*(1,164) = 10.69, *p* = .001, *R*^*2*^_*p*_ = .06, and people who indicated to be religious gave overall higher ratings, *F*(1,164) = 4.58, *p* < .04, *R*^*2*^_*p*_ = .03. Probing the interaction yielded that all simple main effects were statistically significant, all *F*s(1,164) > 22.51, all *p*s < .001, all *R*^*2*^_*p*_ > .12: U.S. Americans were perceived as more prototypical regarding North America than regarding the world in general, and in North America as more prototypical than were non-U.S. Americans; non-U.S. Americans were perceived as more prototypical regarding the world in general than regarding North America; and, in the world in general, they were regarded as more prototypical than U.S. Americans. This successful manipulation of prototypicality provided a good base for testing our substantial hypotheses.

#### Threat

**Fig 3:**
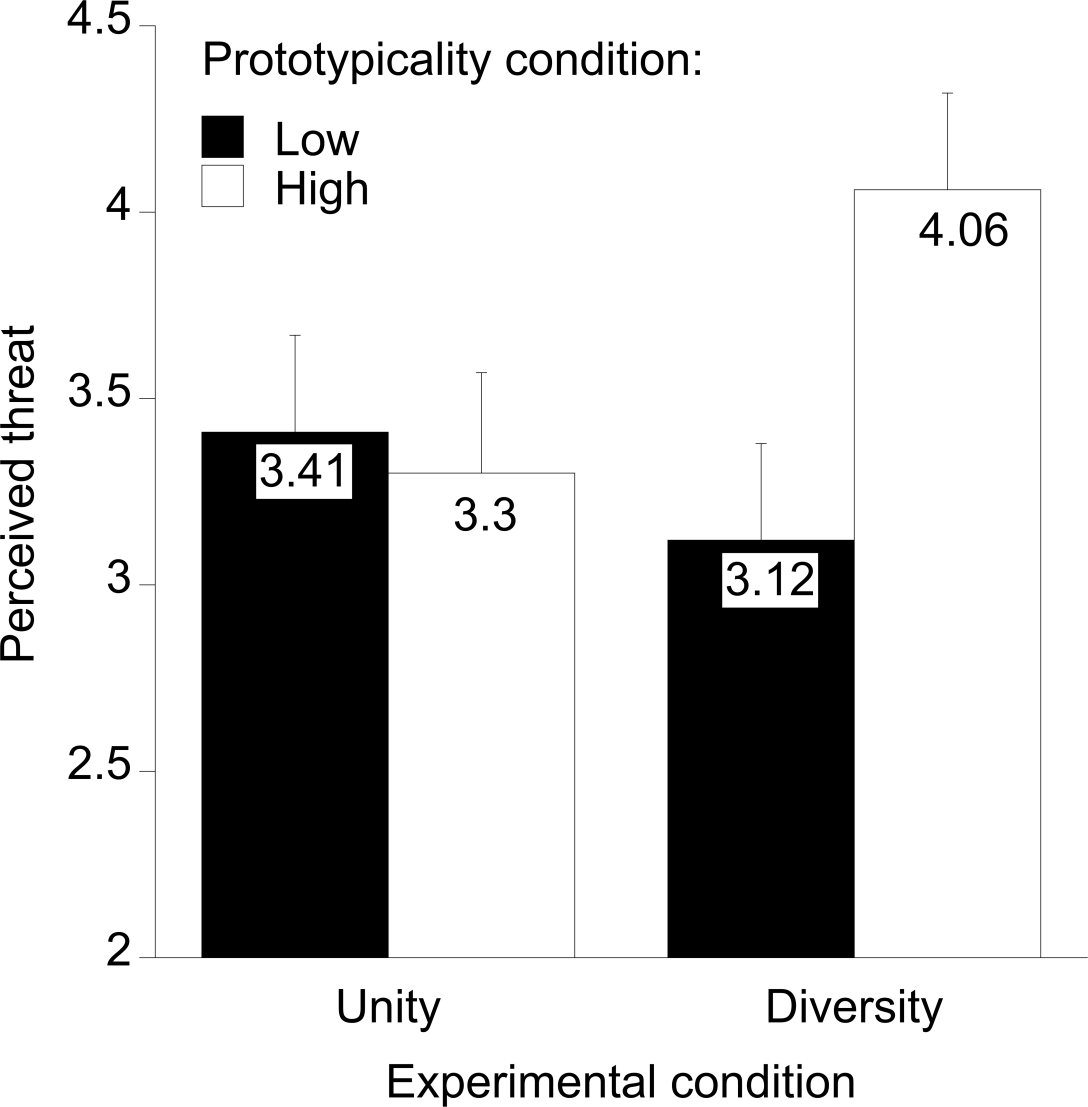
Experiment 3: The effects of the experimental condition (activating diversity vs. unity of the superordinate category) on U.S. Americans’ perceived threat, depending on the context (North America vs. world).

Fig 3 shows perceived threat as a function of condition. It appears that perceived threat is higher in the high prototypicality × diversity condition than in the other conditions. Confirming Hypothesis 1, a 2 (experimental condition: unity vs. diversity) × 2 (context: North America vs. world) ANOVA yielded the expected statistically significant interaction of both factors, *F*(1,165) = 3.96, *p* < .05, *R*^*2*^_*p*_ = .02 (both main effects: *F*s < 2.55). This interaction was due to two significant simple main effects: Corroborating H2, in the diversity condition, threat was higher in the context of North America than in the context world, *F*(1,165) = 6.43, *p* = .01, *R*^*2*^_*p*_ = .04. Also, in the context of North America, threat was higher in the diversity than the unity condition, *F*(1,165) = 4.10, *p* = .04, *R*^*2*^_*p*_ = .02. No other simple main effects were statistically significant (both other *F*s < 1). Of less interest to our purposes, religious U.S. Americans perceived more threat overall than non-religious ones, *F*(1,165) = 13.18, *p* < .001, *R*^*2*^_*p*_ = .07.

#### Outgroup attitudes

Overall, outgroup attitudes were quite positive (*M* = 5.82, *SD* = 1.15, on a 1–7 scale). As in Experiment 1, we tested whether there are indirect effects of threat on outgroup attitudes, depending on the experimental condition and moderated by the context, again following [59]. We found an interaction effect of context condition and experimental condition on threat (replicating the ANOVA finding), *B* = .26, *SE* = .13, *t* = 1.99, *p* < .05. We also found an effect of threat on outgroup attitudes, *B* = -.17, *SE* = .05, *t* = –3.34, *p* = .001. In the context of North America, in line with H3, the indirect effect of experimental condition via threat on outgroup attitudes was statistically significant, *B* = –.06, *SE* = .04 (bias corrected 95%-CI, using 10,000 bootstrapping re-samples:–.17;–.01). The direct effect was not statistically significant, and no indirect effect of experimental condition on outgroup attitudes was observed in the context of the world. In other words, in the context of North America, findings are in line with the idea that people in the diversity condition perceived more threat than those in the unity condition, and this led to less positive outgroup attitudes among them.

Conversely, we entered diversity condition as the moderator and context as the independent variable in a comparable analysis of indirect effects. In the unity condition, there was no direct effect of context condition on outgroup attitudes, nor an indirect effect. In the diversity condition, again in line with H3, the indirect effect of context condition via threat on outgroup attitudes was statistically significant, *B* = –.08, *SE* = .04 (bias corrected 95%-CI, using 10,000 bootstrapping re-samples:–.19; –.02). In other words, these findings are in line with the following interpretation: Within the diversity condition, people in the context of North America perceived more threat than those in the context world, and this led to less positive outgroup attitudes among them.

### Discussion

Experiment 3 provided an important missing piece: An experimental manipulation of the supposed moderator, prototypicality, yielded the expected results: In a superordinate-category context in which the ingroup (U.S. Americans) was high in prototypicality and the outgroup was low (i.e., in the context of North American), activating diversity increased perceived threat. No such effect was observed in a superordinate-category context in which the same ingroup was low in prototypicality and the outgroup was high (i.e., in the context of the world population). This strongly supports the hypothesis that prototypicality is indeed a crucial moderator variable, and not, for instance, a correlate of some other moderator.

Our findings imply that there is a way in which prototypicality perceptions can be manipulated without inducing threat. Specifically, it appears that threat can be avoided by manipulating the social context, that is, the reference group on a superordinate level and thus the ingroup’s status within this context. Counter-intuitively, but in line with our predictions, perceived threat was higher when thinking about the diversity of North Americans than when thinking about the diversity of the world population.

The hypothesis of indirect effects via threat on outgroup attitudes was supported (replicating the preliminary finding in Experiment 1). In the high-prototypicality context (North America), diversity activation led to threat and thus to less positive outgroup attitudes. Similarly, when diversity was activated, outgroup attitudes were more negative in the context of North America than in the context world because threat was increased in the context of North America. This data pattern resembles the original findings that supported the prediction of the ingroup-projection model: that a complex prototype reduced intergroup bias [31]. Those authors also found an indirect effect similar to what we reported in the present experiment and (preliminarily) in Experiment 1.

A drawback of Experiment 3 is that the threat-effect was found on a single-item measure instead of an established scale. Future research should test the generality of this effect on different aspects of threat.

## General discussion

The present experiments tested under which conditions activating superordinate-category diversity improves intergroup relations–or impairs them. According to the ingroup-projection model [18], when focusing on the diversity of the group, the tendency of thinking that all of them are like us should be reduced. However, a more diverse representation of the superordinate category implies a more equal status of the included groups, and the majority may perceive this as a status loss, therefore resenting it [19, 20]. Importantly, in the tradition of research from a social-identity perspective, these general processes are predicted to occur in different intergroup contexts, be they based on sexual orientation, nationality, ethnicity, or on other social identities that people value.

The current studies support our hypothesis that the consequences of activating diversity may depend on perceived prototypicality of the ingroup relative to the outgroup. In detail, activating diversity induced threat in participants who deemed the ingroup as much more prototypical than the outgroup, and it thus impaired these participants’ outgroup attitudes. In contrast, activating diversity reduced threat in participants who considered the ingroup as only a little more prototypical than the outgroup, and it tended to improve these participants’ outgroup attitudes. These findings were corroborated when we manipulated prototypicality perceptions by activating one social context in which participants considered their group as highly prototypical or another in which they thought their group was low in prototypicality. As an important side note, in contrast to the model’s classic assumption, we found that ingroup projection is unaffected in some intergroup contexts by activating the diversity of the superordinate category. By implication, relative ingroup prototypicality is not only an outcome variable, as the ingroup-projection model originally assumes, but can also be an important moderator.

### Implications for the ingroup-projection model and for intergroup-relations research

These findings call for an extension of the ingroup-projection model. Whereas activating diversity, leading to a complex superordinate-category representation, was initially suggested to result in tolerance [18] and first empirical findings were in line with this prediction [23, 31, 32], other findings have hinted at potential moderator variables [44, 66, 67]. The present findings are in line with the analysis we presented in the introduction of this paper that one such moderator is whether one perceives to belong to an ingroup that essentially represents the superordinate category, or not. Participants who perceived their group to be *the* majority showed threat-related reactions and increased intergroup bias when the diversity rather than unity of the superordinate category was activated. Similar findings have recently been observed in a very different intergroup context, concretely, that of metal music fans [68]. Conceptually replicating the present Experiment 2, death metal fans who regarded their ingroup as highly prototypical for metal fans showed more negative attitudes towards a minority group of metal fans after the diversity of the superordinate category had been activated. These and the present findings on prototypicality perceptions, threat, and attitudes are in line with the idea that majority group members try to affirm their group’s position as a reaction to the diversity activation. This points at instrumental processes involved in ingroup projection [69]. In line with this, Rosa and Waldzus [70] demonstrated high levels of ingroup projection when intergroup relations were insecure and participants had the opportunity to systematically process information. Apparently, the threat induced through an insecurity manipulation (the potential loss of a high group status) motivated participants to use “defensive projection” (p. 670). Possibly, among members of large majority groups, thinking about the diversity of the group suffices as an insecurity induction and results in defensive projection. These findings, along with those we presented above, demonstrate that short-term manipulations supposed to reduce ingroup projection do not always work as intended. Whereas thinking about the diversity of the superordinate category is a manipulation that works in some contexts [23, 31, 33], for large majority group members such as those investigated here, more extended interventions may be needed to obtain the aim of reducing ingroup projection [32].

We speculate that activating diversity is a fruitful route towards improving intergroup bias to the degree to which ingroup projection is a heuristic process occurring because of the absence of a clear representation of the superordinate category [22, 67, 71, 72]. As an example, with no clear idea of what the world population is like, as a default one thinks of those who are like one’s own group, which results in ingroup projection [33]. Drawing attention to the diversity within the group, one is reminded of other subgroups belonging to the world population, and in turn, ingroup projection is reduced (also see [70]).

Some other research on intergroup relations has indicated that there may be negative consequences of activating the diversity of the superordinate category. For example, highlighting a common national ingroup with immigrants had positive effects on attitudes towards immigrants in Canada, a country with a long-term supporting policy of multiculturalism [66]. In contrast, in that study negative effects emerged in Germany, where immigration is characterized by segregation or assimilation policies that convey the impression of autochtonous Germans as “real” Germans. Further, the present findings are in line with those demonstrating that activating a diverse representation of the ingroup can bring about undesired costs [73, 74]. Also related are recent findings in the frame of the common ingroup-identity model, suggesting that subgroups view the superordinate category very differently, and consequently, when outgroup members invoke common identity, this may have negative outcomes on intergroup relations [75]. A common thread of this research is that the idea of group diversity may appear threatening [76]. Indeed, a crucial question for practitioners conceptualizing diversity training is how to avoid a backlash effect against diversity programs in members of historically advantaged groups [77]. In other words, there is a growing awareness that a focus on the diversity of a given (superordinate) group may not be welcomed by (sub-)group members who perceive their ingroup to be prototypical for that group [78].

The ingroup-projection model postulates that perceiving the ingroup as *relatively* more prototypical than the outgroup drives negative outgroup evaluations. A relative prototypicality measure was therefore used in our main analyses and consistently functioned as a moderator variable in Experiments 1–3. As a review of findings has shown, often, but not always, outgroup evaluations are more strongly related to the outgroup’s perceived lack of prototypicality than to the ingroup’s high prototypicality [19]. In other words, the outgroup’s lack of prototypicality for the inclusive category often drives effects of relative prototypicality (e.g, Germans’ perception that Italians are not as disciplined as they think Europeans should be). Supplementary analyses of Experiment 1 also showed that the outgroup’s lack of prototypicality drove effects more than the ingroup’s high prototypicality (see Appendix 1). However, in Experiment 2, both ingroup and outgroup prototypicality equally contributed to the observed effects of relative prototypicality. It is presently an open question why these different patterns were obtained in comparable intergroup contexts. Possibly, ingroup prototypicality is less well-established and more varied in adolescents (Exp. 2) than in adults (Exp. 1).

### Can research on multiculturalism profit from the ingroup-projection model?

A line of research that fruitfully connected social-psychological theorizing on intergroup relations with “lay theories” [79] of inter-ethnic relations has examined the consequences of a multicultural ideology for intergroup bias [80]. This research has demonstrated many positive effects of a multicultural ideology (e.g. [9, 10–12, 81, 82]). On top of that, a multicultural orientation is preferred to other ideologies by minority participants [12, 81, 83, 84]. Nevertheless, more recent research demonstrated negative consequences of a multicultural ideology in some or all participants [15, 40, 85]. It was even shown that different ways of activating multiculturalism had opposite effects on perceived threat, outgroup attitudes, and the willingness to engage in intergroup contact [41]. These conflicting findings may draw attention to the fact that it is important to situate research on intergroup relations within the larger social, historic, and cultural context [8, 66, 86]. To obtain this aim, as we argue here, it could help to carry out research on the multiculturalism ideology within established social-psychological theorizing.

Verkuyten [12] was the first to examine multiculturalism using a social-identity approach. We strongly encourage future research on intergroup ideologies to draw on this established theoretical framework and take into account the variables identified as crucial within respective models. Can the ingroup-projection model provide a fruitful framework for research on multiculturalism? Central tenets of multicultural ideology are that differences among groups are valued and celebrated [8], and that society as a whole should become more diverse by accommodating ethnic minority cultures [12]. This can certainly be mapped onto a complex prototype or a complex ideal prototype [25]: If speaking English or French, being White or Asian or Black is all considered prototypical of Canadians [66], then the prototype of this group is complex. Now, the ingroup-projection model goes beyond a multicultural approach in drawing attention to the fact that this can be a route towards tolerance if subgroups are nested within a positively evaluated superordinate category with which they identify. The importance of this specification can be illustrated with reference to the German national situation where in recent years politicians have claimed the failure of “Multikulti” (German for multiculturalism) (see [17] for details). In our view, a reason for this apparent failure is that the creation of a common superordinate category has been neglected (also see [86] for discussion). Turks and other southern Europeans were welcomed as guest workers in Germany in the 1950ies and 1960ies. Being seen as guest workers who would leave eventually (so the initial conceptualization), they were welcomed to stick to their traditions and cultures; but forming a more complex, inclusive German society, integrating them and adapting to them, was omitted. Even though the percentage of immigrants, in relation to the country population, exceeds that of Canada, according to official policy, Germany was not seen as an immigration country (see [87] for details). The term “Ausländer” (foreigner) is generally used to refer to immigrants [87], and even third-generation Turkish immigrants are often treated as foreigners [also based on restrictive naturalization laws that do not follow an ius solis principle of birthplace (as in the U.S.A.), but an ius sanguinis principle of ancestor decent]. Even though they may decide to choose German instead of Turkish citizenship, the established term to refer to Germans with ancestors from Turkey is “Deutsch-Türken” (German Turks), giving primacy to them being Turks. In a nutshell, a social-identity approach to the multiculturalism ideology draws attention to a range of factors that research on intergroup relations should take into account.

Whereas we believe that the ingroup-projection model is a fruitful framework for research on the multiculturalism ideology, we concede that multiculturalism is too unspecified to map only onto one of the models developed within the social-identity approach (see also the findings by [41]). For example, recent formulations of the common ingroup-identity model, stressing dual identities, can also serve as a theoretical basis to analyze multiculturalism [88]. In fact, rather than being likened to a multicultural ideology, a complex representation of the superordinate category is most similar to *all-inclusive multiculturalism*, emphasizing that diversity includes both minorities and majorities alike [74, 89].

Relating the color-blindness ideology to psychological theorizing gets even more ambiguous. “The color-blind model emphasizes the sameness of people, that racial categories should be ignored or avoided, and that differences based on social identity should be assimilated into an overarching unifying category” ([8], p. 85). This appears well in line with a social-identity approach according to which ingroup-outgroup categorization is the starting point for discrimination [2], and thus, avoiding such categorization may reduce intergroup bias. But should the color-blind ideology be likened to higher-level categorization within a common ingroup [88]? Or to de-categorization, treating everyone as a unique individual [90]? Even though preserving “the preference for unity” ([89], p. 120), we believe color-blindness does not include the notion of a simple prototype as specified in the ingroup-projection model because a simple prototype appears more in line with an assimilation ideology, “the adoption of majority-group cultural characteristics as the basis” for the common identity ([91], p. 208).

This vagueness of the lay theories strengthens our argument that well-tested social-psychological theoretical approaches may serve as a better basis for empirically investigating diversity. Importantly for the aim of the present paper, research on lay ideologies is in line with our idea that drawing participants’ attention to diversity within a superordinate category may either provoke a complex prototype, or it may be met with resistance in highly-prototypical majority members. Future research should aim to identify the conditions under which these participants do not feel threatened, but also adapt their prototype and embrace diversity (for potential starting points, see [74, 92]). As compared to a mere priming message, an extended training experience, consisting of different modules and lasting several hours, may yield more promising findings [32]. In sum, a broader level of theorizing, looking for general principles within the specificities relevant in a given intergroup situation, relying on detailed theories and models, may be more fruitful for testing how to improve intergroup relations than a short ideological message separated from larger theorizing.

### Limitations, future research perspectives, and practical implications

Several limitations need to be mentioned. Ingroup identification did not moderate the present findings, even though identification has been a moderator variable in previous research on ingroup projection ([93], but see [27]). One reason for this could be that we used groups in our research with which most participants indicate high identification (e.g., being a man). Future research is needed to integrate findings on prototypicality and identification as potential moderator variables (see [94] for an analysis of the conceptual differences between identification and prototypicality).

A second limitation we need to mention is that we relied on several single-item measures (attitudes in Exp. 1, threat in Exp. 3) and on scales that are not yet well-validated, but were constructed for the present purposes (the threat measure in Exp. 1, attitude measures in Exp. 2–3). The questionable validity of some of our measures could be a reason why the effects we found tended to be smaller than expected. The generality of the present findings should be tested using more extended and validated measures and larger samples.

The consistency of our findings across gender and nationality intergroup contexts corroborates the social-identity perspective’s assumption that similar processes operate in all intergroup contexts if people identify with the groups in question. However, such intergroup contexts still differ in important structural features, and findings could be moderated by such differences. In addition to the relationship between ingroup and superordinate category that we investigated here, there are other aspects of the intergroup relation that could play a role when predicting the consequences of a diversity activation: Features pertaining (i) to the outgroup, (ii) to the superordinate category, and (iii) to the ingroup. Examining features of the *outgroup* that may affect reactions to a diversity activation is related to the following questions: Is the status of the outgroup high or low? What is its relation with the ingroup? Is the outgroup perceived as included in the superordinate category, or not? For instance, within the European context, the effects of a diversity manipulation on reactions towards Poles (included in the European Union) versus Turks (not included) may differ. Moreover, is the outgroup perceived as a subgroup or a subtype [54]? Does its exclusion appear legitimate? Further, both *superordinate categories* and *ingroups* may differ with respect to their complexity (e.g., Europeans could per se be represented as a more complex group than Germans) and their entitativity [95]. In addition, the defining features of the group could be perceived as essential or changeable, and the group’s diversity could be valued or evaluated negatively to begin with [96]. Moreover, the superordinate category and the ingroup may be relatively chronic ones with which identification is generally high and that are central to the self. All of this may prove to moderate the consequences of activating diversity. We consider it worthwhile to put these factors to empirical tests in order to increase knowledge on the important question for whom, under which circumstances, drawing attention to within-group diversity has positive consequences.

From a practical stance, an approach to positive intergroup behavior that is currently en vogue in organizational contexts is diversity management, intended to exploit the potential of social diversity within the workforce in order to provoke competitive benefits [97]. A main aim of the respective efforts is that social diversity within a superordinate group (e.g., an organization) be seen as a virtue rather than a vice. Whereas the practice of diversity management does not overly rely on social-psychological models [77] and the causal effects of prejudice-reduction interventions such as diversity training remain largely unknown [98], the ingroup-projection model appears well suited as a theoretical base for such interventions. The present research points out that analyses are timely that specify for which group members diversity interventions appear suited. There are group members who cling to the status quo, be it the prototypical ones (as in the present research); authoritarians [40, 99] or those with high conservation values [73]; those who do not value diversity [96]; or political conservatives [41]. For these group members, other means than drawing attention to the social diversity of the group may turn out to be more fruitful for improving intergroup relations [14, 77].

## Conclusion

In the face of growing multiculturalism in many societies, in schools, and in organizations, the present research deals with a timely theoretical and practical problem: How can we design interventions in order to obtain that social diversity within a superordinate group is valued? The ingroup-projection model provides useful theoretical underpinnings for such interventions. However, as we have demonstrated, drawing attention towards group diversity, a measure that has been shown to be fruitful for some individuals [23, 31, 32], is highly problematic for others (also see [14, 73]). We hope to have inspired other researchers to tackle the open questions raised in the previous paragraphs. What we have identified so far is that drawing attention to group diversity may backfire in those individuals considering their ingroup as highly prototypical for the superordinate category—just the ones who are the ‘prototypical’ targets of prejudice-reduction interventions.

## Appendix 1

Separate analyses of ingroup and outgroup prototypicality following [100].

## Experiment 1

*Moderation of the effect of diversity activation by prototypicality*. We computed interaction terms between condition (–1: unity, 1: diversity) and the z-standardized ingroup prototypicality and outgroup prototypicality scores. Experimental condition, ingroup prototypicality, and outgroup prototypicality were entered simultaneously as predictors of *general threat* and *other-group threat*, respectively, in multiple regression analyses. No statistically significant interactions were found on *general threat*, *β*s = .10, *p* > .30, nor were the overall regression models significant (*F*s < 1.04).

For *other-group threat* as the dependent variable, the overall regression model without the interaction was statistically significant, *R*^*2*^ = .11, *F*(3,93) = 3.90, *p* = .01. Experimental condition did not affect threat, *B* = .01, *SE* = .11, *β* = .01, *|t|* < 1, nor did perceived ingroup prototypicality, *B* = –.08, *SE* = .11, *β* = –.08, *|t|* < 1, whereas participants who perceived the outgroup to be less prototypical for the superordinate category reported significantly more threat than participants perceiving higher outgroup prototypicality, *B* = –.32, *SE* = .11, *β* = –.30, *t* = –2.90, *p* = .005. More central to H1, with the interaction terms entered in the second step of the regression, there was a significant increase in explained variance in other-group threat, Δ*R*^*2*^ = .08, Δ*F*(2,91) = 4.66, *p* = .01. Whereas ingroup prototypicality did not significantly modify the relationship between experimental condition and other-group threat, *B* = .10, *SE* = .11, *β* = .09, *|t|* < 1, perceived outgroup prototypicality did, *B* = –.33, *SE* = .11, *β* = –.31, *t* = –3.05, *p* = .003.

To assess the nature of the interaction, simple slopes were computed. Among participants perceiving gay men to be rather prototypical for men in general (1 *SD* above the mean), the slope was *B* = –.29, *SE* = .14, *β* = –.27, *t* = –2.01, *p* < .05. This significant effect indicated that participants who perceived gay men as rather prototypical of men in general experienced less threat in the diversity compared to the unity condition. The effect was reversed among participants perceiving the outgroup to be low in prototypicality for the superordinate category (1 *SD* below the mean), *B* = .31, *SE* = .14, *β* = .29, *t* = 2.16, *p* = .03, indicating that they perceived significantly more threat in the diversity compared to the unity condition (confirming H2).

*Outgroup attitudes and moderated mediation*. We next tested whether there was an effect of the experimental condition on attitudes, mediated via threat, and whether this mediation is moderated by perceived outgroup prototypicality [following 61]. We allowed the effect of experimental condition on the mediator other-group threat to be moderated by perceived outgroup prototypicality. Replicating what we report above, there was no main effect of experimental condition on perceived threat, but both an effect of perceived outgroup prototypicality and an interaction between both variables. The regression analysis in which outgroup attitudes were regressed on experimental condition and threat yielded only a significant effect of threat, *B* = 0.24, *SE* = 0.06, *t* = 3.92, *p* < .001 (experimental condition, *|t|* < 1). A bootstrapping analysis with bias-corrected confidence intervals and 10,000 bootstrap re-samples confirmed that there was no direct effect of experimental condition on outgroup attitudes (*B* = –0.06, *SE* = 0.06, *|t|* < 1). For participants who perceived the outgroup as low in prototypicality (–1 *SD* below the mean), thinking about diversity induced threat and thus decreased outgroup attitudes (i.e., indirect effect of experimental condition via threat on outgroup attitudes, confirming H3: *B* = 0.07, *SE* = 0.04; 95% *CI*: 0.006; 0.15). For participants who perceived the outgroup as high in prototypicality (+ 1 *SD*), thinking about diversity reduced threat and thus made outgroup attitudes more positive (H4, *B* = –0.06, *SE* = 0.03; 95% *CI*: –0.15; –0.01). No evidence of mediation was found at the mean of the moderator variable.

In sum, these findings demonstrate that the effects reported for relative prototypicality in the main text are based on outgroup, not ingroup, prototypicality.

## Experiment 2

Note that the sample size is small considering the number of predictors in the regression model reported below.

*Moderation of the effect of diversity activation by prototypicality*. Controlling for participant gender, we tested whether attitudes towards lesbian girls or gay boys, respectively, depended on experimental condition (–1: unity; 1: diversity) and centered ingroup and outgroup prototypicality scores.

The regression model was statistically significant, *F*(4,70) = 6.71, *p* < .001, *R*^*2*^ = .28. Lower perceived prototypicality of lesbian girls or gay boys was related to more negative attitudes, *B* = –.47, *SD* = .17, β = –.31, *t* = –2.78, *p* < .01, and boys’ attitudes towards gay boys were more negative than girls’ attitudes towards lesbian girls, *B* = .79, *SD* = .33, β = .26, *t* = 2.40, *p* < .02, but there was no main effect of experimental condition, *|t|* < 1 (see ANOVA in the main text). Higher perceived prototypicality of heterosexual girls or boys was associated non-significantly with more negative outgroup attitudes, *B* = .27, *SD* = .16, β = .18, *t* = 1.72, *p* = .09. In Step 2, adding the interaction terms significantly increased the explained variance in attitudes, Δ*F*(2,68) = 3.42, *p* < .04, Δ*R*^*2*^ = .07. However, both the interaction of experimental condition and outgroup prototypicality, *B* = –.26, *SD* = .15, β = –.17, *t* = –1.66, *p* = .10, and the interaction of experimental condition and ingroup prototypicality, *B* = .26, *SD* = .15, β = .17, *t* = 1.68, *p* = .09, were non-significant given the sample size.

In sum, these findings demonstrate that the effects reported for relative prototypicality in the main text are based partly on outgroup and partly on ingroup prototypicality.

## Appendix 2

English translations of items used to measure other-group threat to the male prototype in Experiment 1:

1. As a man I feel uncomfortable if I think about other men influencing the image of men by acting out their sexuality.
2. If women these days appreciate “soft” traits in men, most heterosexual men will lose.
3. It does not bother me that women as a rule find gay men attractive. (R)
4. Gay men provoke an inacceptable deterioration of established roles and norms.
5. I feel uneasy if I think of the traits that I as a man potentially lack.

Original German items:

1. Ich als Mann fühle mich unwohl, wenn ich daran denke, wie manche Männer durch ihre Form der ausgelebten Sexualität mein Männerbild beeinflussen.
2. Wenn Frauen heutzutage vermehrt auf „weiche”Eigenschaften bei Männern Wert legen, können die meisten heterosexuellen Männer einpacken.
3. Der Gedanke, dass Frauen an sich homosexuelle Männer attraktiv finden könnten, läßt mich kalt. (R)
4. Schwule Männer führen zu einer nicht vertretbaren Aufweichung gängiger Rollen und Normvorstellungen.
5. Mir wird bange, wenn ich an all die Eigenschaften denke, die mir als Mann möglicherweise fehlen könnten.

## Supporting information

S1 File SPSS data file providing the raw data of experiment 1. (SAV)

S2 File SPSS data file providing the raw data of experiment 2. (SAV)

S3 File SPSS data file providing the raw data of experiment 3. (SAV)
